# Gastroprotective and gastric motility benefits of AD-lico/Healthy Gut™ *Glycyrrhiza inflata* extract

**DOI:** 10.1080/19768354.2017.1357660

**Published:** 2017-08-18

**Authors:** Ali Sadra, Hyuck-Se Kweon, Sung-Oh Huh, Jaeyoung Cho

**Affiliations:** a Department of Pharmacology, College of Medicine, Hallym University, Chuncheon, Gangwon-Do, South Korea; b SynergyBio, Chuncheon, Gangwon-Do, South Korea; c ADbiotech Co. Ltd., Chuncheon, Gangwon-Do, South Korea; d Unites Inc., Chuncheon, Gangwon-Do, South Korea

**Keywords:** Licorice extract, *Helicobacter pylori*, stomach comfort

## Abstract

The aim of this study was to evaluate *in vivo* both the anti-*Helicobacter* and the gastric-relaxing effects of AD-lico/Healthy Gut™ in rat models. AD-lico/Healthy Gut™ is a specially prepared commercial formulation of *Glycyrrhiza inflata* extract that is under clinical development for indications of gastrointestinal disease and inflammatory bowel disease. In the current study, the oral administration of AD-lico/Healthy Gut™ significantly reduced mucosal damage from *Helicobacter pylori* in rats and decreased the expression of the inflammatory markers iNOS and COX-2 in the test cells. AD-lico/Healthy Gut™ also reduced mucosal damage caused by water immersion stress in rats. The accelerated gastric emptying in normal rats was also seen with AD-lico/Healthy Gut™, providing relief in gastric relaxation in the test animals. The special formulation of AD-lico/Healthy Gut™ with reduced levels of component glycyrrhizin also has benefits in minimizing the potential side effects attributed to glycyrrhizin seen with similar *Glycyrrhiza* extracts in terms of induction of hypokalemia and muscle weakness. The preparation has a relatively high phenolic compound content relative to other methods of preparation and is indicative of lower glycyrrhizin levels. These results suggest that AD-lico/Healthy Gut™ may provide the necessary relief from a number of stomach discomfort issues faced by a large population of people.

## Introduction

As a Leguminosae perennial, licorice plant is cultivated in many countries including parts of Asia. Licorice is comprised of about 30 species containing the *Glycyrrhiza* genus (Karkanis et al. 2016), and it has been used as a traditional medicine for various benefits such as anti-inflammatory, antibacterial, anti-viral and palliative effects (Wang et al. 2015). The ethanol extracts from the licorice root have been in use as alternative medicine for patients with various stomach maladies such as gastric and duodenal ulcers.

Licorice root has about 300 flavonoids and 20 triterpenoids. These include 73 bioactive components and 91 potential targets that have been identified and isolated (Li et al. 2011, 2013). These bioactive components include liquiritigenin, licochalcone E, glycyrrhizin (GL), glabridin and licochalcone A that have been most researched for their activities. Certain licorice extracts, however, have only been used in limited applications and doses due to serious adverse effects presented. These adverse effects have been identified as being mostly due to GL as overconsumption of licorice containing more than 2 mg/kg/day of pure GL may result in cases of muscle weakness and hypokalemia (Omar et al. 2012). These properties of GL are possibly due to its inhibitory effect on cortisol degradation, leading to weight gain or weight loss, edema, hypertension and hypokalemia (Armanini et al. 2002). We have had success in isolating licorice extracts containing very low levels of GL and higher levels of beneficial phenolic compounds than conventional licorice extracts. AD-lico/Healthy Gut™ is a commercial 95% ethanol extract from *Glycyrrhiza inflata* and it is under clinical development for gastrointestinal diseases including inflammatory bowel disease.

*Helicobacter pylori* is the major target species of bacteria that routinely infects the stomach. It has been shown to cause various stomach maladies that include peptic ulcer disease, gastritis, gastric adenocarcinoma and mucosa-associated lymphoid-tissue lymphoma. *H. pylori* is also recognized as a Class-1 carcinogen by the International Agency for Research on Cancer (IARC) (Manyi-Loh et al. 2010). *H. pylori* is also recognized as a virulent pathogen, requiring a low infective dose and having a high prevalence of 20–50% in industrialized countries and in excess of 80% in developing countries (Ndip et al. 2004). *H. pylori* is difficult to treat, having a relatively high treatment failure rate of 10–40% (Lai et al. 2006). One reason for the treatment failure is the growing resistance of *H. pylori* to mainstream antimicrobials given to patients (Tanih et al. 2010). This is demonstrated by the shocking 100% resistance reported against metronidazole, one of the drugs in the treatment regimen in certain countries in the developing world (Aboderin et al. 2007). A reduction in the effectiveness of antibiotics therapy against *H. pylori* also includes the relatively high cost of combination therapy, in addition to drug side effects and poor patient compliance (Romano and Cuomo 2004). As such, developing new anti-*Helicobacter* agents is of great importance and new modes of therapy to eradicate or combat the effects of *H. pylori* infection are required. These may include the development of vaccines, use of probiotics and nutraceuticals such as various bioactive extracts (Ayala et al. 2014).

For this study, the anti-*H. pylori* properties of AD-lico/Healthy Gut™ are demonstrated in rats in terms of improvement in a dose-dependent manner of mucosal damage caused by the pathogen. Also in this study, the benefits of AD-lico/Healthy Gut™ in improving stomach function were investigated in an animal model of delayed gastric emptying (GE) (Tack et al. 2004; Mimidis and Tack 2008).

The symptoms of GE usually include individuals with functional dyspepsia (FD), which is a chronic gastrointestinal disorder, causing considerable discomfort to the patient (Mimidis and Tack 2008). Although the causes of FD are not well understood, it is associated with pathophysiologic changes that include delayed stomach emptying and gastric accommodation. There is also observed visceral hypersensitivity (Mimidis and Tack 2008). In all, 30–40% of FD patients have delayed GE (Talley et al. 2006), with an approximately 40% of FD patients also having impaired gastric accommodation after meals (Tack 2000). In this study, the rat model of GE demonstrated AD-lico/Healthy Gut™ having a benefit with accelerated GE in normal rats and providing relief in gastric relaxation in the test animals. This validated the potential use of this extract in alleviating the symptoms of GE in individuals experiencing it.

Both the anti-*Helicobacter* and stomach emptying properties of AD-lico/Healthy Gut™ indicate a potential in addressing stomach discomfort in a sizable population. The low levels of component glycyrrhizin coupled with a relatively high phenolic compound content for AD-lico/Healthy Gut™ are also indicative of lower possible side effects associated with the use of this product. As mentioned above, AD-lico/Healthy Gut™ is also under clinical development for gastrointestinal diseases including inflammatory bowel disease.

## Materials and methods

### AD-lico/Healthy Gut™ source material and *H. pylori* stock

AD-lico/Healthy Gut™ used in these experiments is a licorice (*Glycyrrhiza inflata*) ethanol extract under development by ADbiotech Co. Ltd. (Chuncheon, Gangwon-Do, Korea). Briefly, the preparation processes for AD-lico/Healthy Gut™ involved steps for licorice root powder roasting, ethanol extraction, concentration, precipitation, washing of the extract powder and drying of the extract powder all under controlled conditions and with quality control. *H. pylori* stock was from the American Type Culture Collection (ATCC) (strain ATCC 43504; human gastric antrum, Australia) (Rockville, MD, USA).

### Measurement of phenolic compounds

The protocol involved using the Folin-Ciocalteu method based on phenolic compounds leading to reduction of a phosphotungstate-phosphomolybdate complex and generating blue reaction products (Vinson et al. 2001). The reaction mixture was reduced to 1 ml and each sample was read at 760 nm and with 30 min of reaction allowed to proceed in a comparison against blank water. The linear calibration curve was generated using gallic acid as the standard, from three replicate wells and allowing an absorbance range of 3 AUs. The total phenolic content values were quantified from the average of three replicate wells and the gallic acid equivalents (GAE) were determined from the curve fir of those average values. The assay allowed for high reproducibility for the standards and samples.

### DPPH radical scavenging assay

The 2,2-diphenyl-1-picrylhydrazyl (DPPH) radical scavenging activity was evaluated as previously described (Gupta and Gupta 2011) and with modifications. In this assay, 0.2 ml of sample at different concentrations (1–1000 μg/ml) was added to 1.8 ml of DPPH solution (0.11 mM) in 80% ethanol. The mixture was then incubated for 30 minutes and at room temperature in the dark. The absorbance at 517 nm was then measured with ascorbic acid as reference antioxidant. A 0.2 ml of solvent dilutant was added to 1.8 ml of DPPH solution (0.11 mM) in 80% ethanol to serve as the negative control. Two independent experiments were performed in triplicate. The percentage inhibition was calculated relative to the control using the equation, inhibition of DPPH radical (%) = (1 − (Abs_sample_/Abs_control_)) × 100. The calculated values are the average of three replicate reads, along with a standard deviation value provided for each.

### *H. pylori* infection in rats

Male Sprague-Dawley rats (Orient Bio Inc., Seongnam, Korea) weighing at 220–250 g were used. To establish *H. pylori* infection, the rats were three times inoculated with 0.8 ml of sterile culture containing 1 × 10^8^ colony-forming units (CFUs) of *H. pylori* via gastric intubation and at 48 h intervals. Prior to the first inoculation, the animals were fasted for 24 h. The protocols on treatment of the laboratory animals were approved by the Institutional Animal Care and Use Committee (IACUC) in the laboratory.

### Pathologic assessment of damage caused by *H. pylori*

Paraffin-embedded tissue was sectioned into 4 μm thickness slices and then stained with hematoxylin and eosin (H&E) to detect for inflammatory and epithelial changes. Infiltration of neutrophils and lymphocytes indicated mucosal damage, and the level of pathologic changes for inflammation, hyperplasia and peptic ulceration was on a 4-point scale (0–3: 0: normal; 1: mild; 2: moderate; 3: marked) and according to the modified and updated Sydney System (Cao et al. 2007).

### Cell culture and *H. pylori* infection

Human gastric epithelial AGS cells (gastric adenocarcinoma, ATCC CRL1739) were from ATCC. They were cultured in RPMI-1640 medium containing 10% fetal bovine serum, 4 mM glutamine, 100 units/ml penicillin and 100 μg/ml streptomycin (GIBCO-BRL, Grand Island, NY, USA). The cells were seeded in six-well culture plates and at the density of 3 × 10^5^ cells per well. They were cultured and used when reaching 80% confluency. Before treatment of the cells, each well was first washed with 2 ml of fresh culture medium with no antibiotics. *H. pylori* from a chocolate agar plate was first suspended in antibiotic-free culture medium with 10% fetal bovine serum and then added to the cultured cells at a ratio of 300:1 (bacterium/cell). This optimal ratio of cells was obtained from a previous study (Cho et al. 2010).

### Western blot for iNOS and COX-2 expression in gastric epithelial AGS cell cultures

AGS cells treated with *H. pylori* were first trypsinized, and then washed. They were then homogenized in lysis buffer made up of Tris–HCl (pH 7.4), 1% NP-40 and cocktail of protease inhibitors (Boehringer Mannheim, Indianapolis, IN, USA). The protein lysate concentration for each sample was determined by the Bradford assay (Bio-Rad, Hercules, CA, USA). Total cell extracts at 50–100 μg were separated with 7–12% SDS polyacrylamide gel electrophoresis under reducing conditions and transferred onto nitrocellulose membranes (Amersham, Arlington Heights, IL, USA) for Western blotting. After blocking with 5% nonfat dry milk for 1 h, the membranes were incubated with COX-2, iNOS and actin antibodies (Santa Cruz Biotechnology, Santa Cruz, CA) in TBS-T containing 5% nonfat dry milk at 4°C overnight. Following washing with TBST (Tris-buffered saline, 0.1% Tween 20), the immunoreactive proteins were visualized by using goat anti-mouse (COX-2), goat anti-rabbit (iNOS) or donkey anti-goat (actin) secondary antibodies conjugated to horseradish peroxidase and visualized by enhanced chemiluminescence (Santa Cruz Biotechnology).

### Mucosal damage assessment after water immersion stress

Stress-induced gastric mucosal damage was induced in rats with a modification of a method described earlier (Takagi et al. 1964; Takagi and Okabe 1968). Normal male Sprague-Dawley rats at 220–250 g (Orient Bio) were restrained in special cages and were then immersed in water at 23°C to the xiphoid level. After being exposed to 3.5 h of water/restraint protocol, the animals were sacrificed, and their stomachs were removed, which were dissected along the greater curvature. The mucosal damage was assessed macroscopically and the number of gastric lesions was noted in each stomach of rat belonging to different experimental groups. The protocols on treatment of the laboratory animals were approved by the IACUC in the laboratory.

### Assessment of GE

GE was assayed according to published protocol (Ozaki and Sukamoto 1999). Normal male Sprague-Dawley rats at 220–250 g (Orient Bio) were fasted for 18 h with ad libitum access to water. The rats were then given 2 ml of semi-solid meals by gavages at 60 min following drug administration. After 30 min, animals were sacrificed, and their stomachs and contents were weighed and photographed in order to determine GE. This protocol was approved by the local IACUC.

### Statistical analysis

Data are presented as mean ± SD. To determine statistical significance, data were analyzed using Student’s *t-*test with Microsoft Excel 2010 program (Microsoft Inc., Redmond, WA, USA). A *p*-value of less than .05 indicated statistical significance.

## Results

### Improved extraction of phenolic compounds from *Glycyrrhiza inflata* licorice root powder

In order to identify the extraction method resulting in maximal levels of phenolic compounds from *Glycyrrhiza inflata,* solvents of varying ethanol content were tested in the extraction protocol. Table 1 shows the quantitation of the phenolic compounds resulting from the various solvents tested. The 95% ethanol extraction solvent gave the highest levels of phenolic compounds from the ground licorice root powder.

**Table 1.** Level of phenolic compounds in various ethanol extracts of *Glycyrrhiza inflata* root powder.

Type of extract	95% Ethanol (50 µg/ml)	80% Ethanol (50 µg/ml)	50% Ethanol (50 µg/ml)	Water (50 µg/ml)
Phenolic content (mg GAE/g of sample)	280	200	170	20

Notes: Isolated licorice extracts contained relatively high levels of beneficial phenolic compounds. The phenolic content measurement was performed on a batch of AD-lico/Healthy Gut™, performed in triplicate and according to the Materials and Methods. The curve-fit values were derived from the average absorbance of triplicate reads. This is a representative of three experiments.

### Radical scavenging activity using the DPPH activity assay

Several diseases, including erosive gastritis and inflammatory bowel disease, are related to oxidative stress (Pavlick et al. 2002). Thus, the antioxidant effect of licorice extracts were compared with ascorbic acid and rebamipide (Zakaria et al. 2014). As the data in Table 2 show, the activity of the extracts is increased by increasing the concentration of ethanol in the extraction. The 95% ethanol extract had the highest level of antioxidant activity among the extracts tested. This is the extract used in the manufacture of AD-lico/Healthy Gut™.

**Table 2.** Antioxidant activity of AD-lico/Healthy Gut™ (DPPH assay).

Radical scavenging activity	AD-lico/Healthy Gut™ (50 µg/ml)	Ascorbic acid (300 µM)^a^	Rebamipide (100 µg/ml)	
DPPH radical scavenging activity, %	90.62 ± 0.35	37.60 ± 0.47	0.93 ± 0.83	
Type of extract	95% Ethanol (50 µg/ml)	80% Ethanol (50 µg/ml)	50% Ethanol (50 µg/ml)	Water (50 µg/ml)
DPPH radical scavenging activity, %	90.59	70.43	54.95	10.62

Notes: Several diseases, including erosive gastritis and inflammatory bowel disease, are related to oxidative stress (Pavlick et al. 2002). Thus, the anti-oxidative effect of a batch of AD-lico was compared with ascorbic acid and rebamipide, a drug approved for mucosal protection, remedying stomach ulcers and gastritis (Matsuda et al. 2003). AD-lico/Healthy Gut™ and various ethanol extracts were tested for scavenging DPPH radical. Ascorbic acid served as the antioxidant standard. The average value of three replicates is provided, including the standard deviation calculation for each. This is a representative of three experiments.

^a^IC50 of ascorbic acid at 50 µg/ml.

### AD-lico/Healthy Gut™ alleviated *H. pylori*-induced mucosal damage

**Figure 1.**
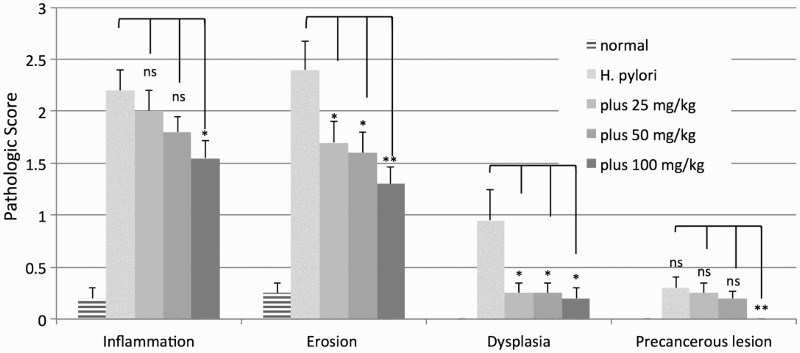
Improvement of dose-dependent mucosal damage caused by *H. pylori* in rats. The variables were normal control animals, *H. pylori* treated animals, *H. pylori* plus AD-lico/Healthy Gut™ 25 mg/kg, *H. pylori* plus AD-lico/Healthy Gut™ 50 mg/kg, and *H. pylori* plus AD-lico/Healthy Gut™ 100 mg/kg. The pathologic scores from the tissue slides were calculated and plotted. The error bars are SD values from five animals per group. This is a representative of two experiments. In the plot, relative to *H. pylori* control, not significant (ns), *p < .05, and **p < .01 were the designations of significance.

As shown in Figure 1, there was a dose-dependent improvement in the mucosal damage caused by *H. pylori* infection in rats. This was based on assessment of paraffin-embedded mucosal stomach samples serially sectioned and stained with H&E for histological analysis for inflammatory and/or epithelial changes. Mucosal damage was defined as infiltration by neutrophils and lymphocytes. The degree of inflammatory change, hyperplasia and peptic ulceration was also graded according to a modified Sydney System (Cao et al. 2007). All the parameters – inflammation, erosion, dysplasia and precancerous lesions – were improved by oral AD-lico/Healthy Gut™. The arms of the experiments were normal rats, *H. pylori*-infected rats and those infected but also having received increasing levels of ingested of AD-lico/Healthy Gut™ licorice extract (25, 50 and 100 mg/kg).

### AD-lico/Healthy Gut™ improved the protein markers of inflammation in AGS cells

**Figure 2.**
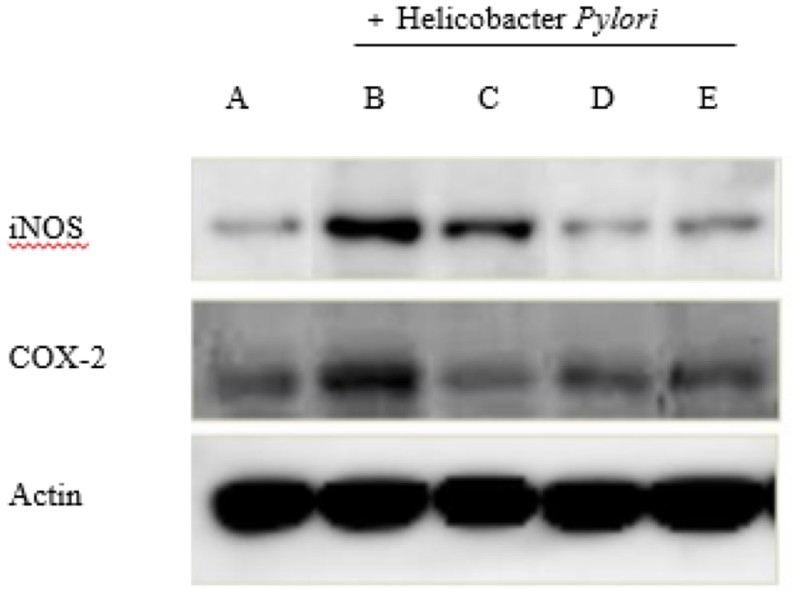
AD-lico/Healthy Gut™ downregulated *H. pylori*-induced iNOS and COX-2 expression in AGS cells. (A) Normal control; (B) negative control; (C) AD-lico/Healthy Gut™ 25 mg/kg; (D) AD-lico/Healthy Gut™ 50 mg/kg; (E) AD-lico/Healthy Gut™ 100 mg/kg. Western blots of the treated cell lysates were probed for iNOS, COX-2 and actin.

Looking at the indicators of inflammation, iNOS and COX-2, via Western blot in AD-lico/Healthy Gut™ treated human AGS gastric adenocarcinoma cells infected with *H. pylori*, there was a significant reduction in the levels of these two markers in the extracts of the treated cells (Figure 2). This indicated that AD-lico/Healthy Gut™ was capable of directly acting on the stomach mucosal cells in reducing the extent of damaging inflammation.

### AD-lico/Healthy Gut™ led to an improvement in mucosal damage caused by water immersion stress in rats

**Figure 3.**
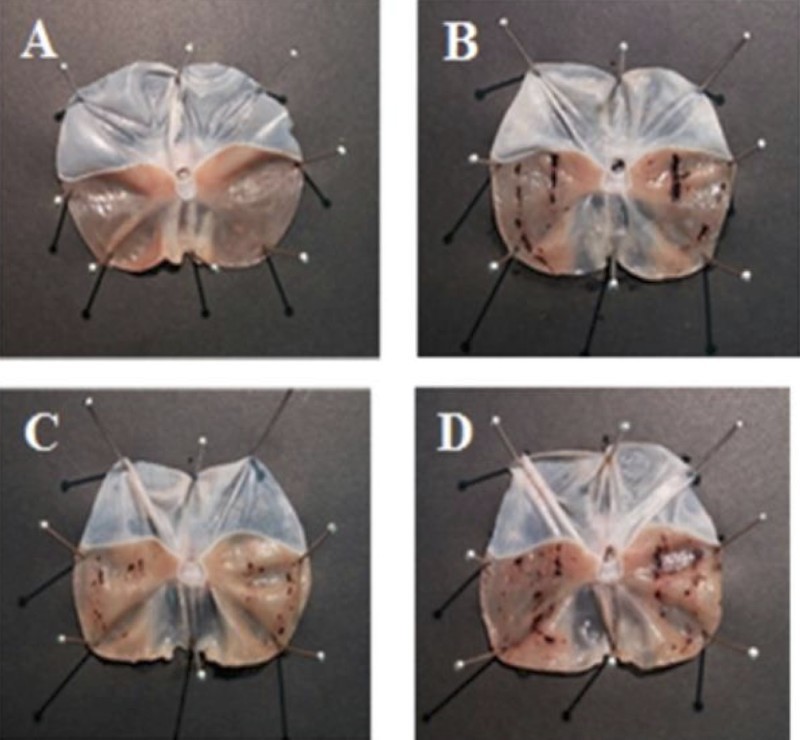
Improvement of mucosal damage caused by water immersion stress in rats with AD-lico/Healthy Gut™. (A) Normal control; (B) negative control; (C) AD-lico/Healthy Gut™ 25 mg/kg; (D) SWG 50 mg/kg. AD-lico/Healthy Gut™ showed better protective effects when compared with an approved functional food (SWG) (Korea) to improve stomach function. After the water/restraint protocol, the animals were sacrificed; their stomachs were then removed, and dissected along the greater curvature of the stomach. Mucosal damage was assessed macroscopically. Representative specimens are shown.

In a model of mucosal damage performed in rats (water immersion test) in Figure 3, oral administration of AD-lico/Healthy Gut™ ameliorated the number of gastric lesions and damage seen visually on stomach surface compared with unstressed rats and those that were stressed, but were untreated. This effect was significantly better than skin white ginseng (SWG), a functional food in Korea recommended to improve stomach function.

### Improvement in GE in normal rats

**Figure 4.**
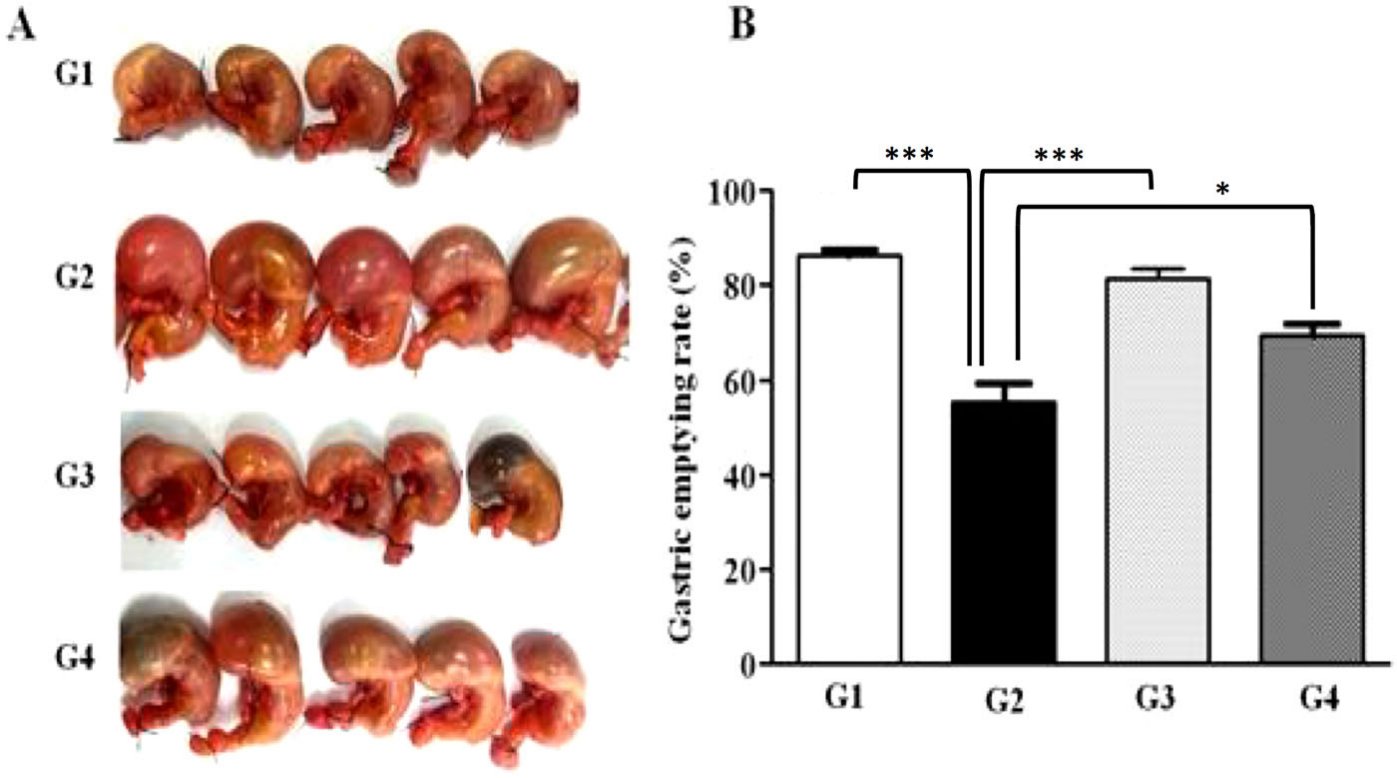
Improvement of GE in rats. G1, Normal control; G2, negative control; G3, mosapride 10 mg/kg; G4, AD-lico/Healthy Gut™ 100 mg/kg. The rats were fasted and then given measured semi-solid meals by gavages at 60 min following drug administration. After 30 min, animals were sacrificed, and their stomachs and contents were weighed and photographed. Five (5) samples per group are shown (A), along with quantitated GE rates (B). ***p < .001 and *p < .05 denote significance compared with G2 negative control.

To identify any potential benefits of AD-lico/Healthy Gut™ in providing relief for stomach emptying, GE was measured according to the Materials and Methods and for post-fasting rats. Figure 4 displays the excised stomach of treated animals post feeding and the quantitation of their GE rate. Following drug administration, when compared with untreated control and 30 min post feeding, AD-lico/Healthy Gut™ treated group had significantly higher GE. This improvement was comparable to mosapride, an improved gastroprokinetic drug (Tack et al. 2012). From this observation, AD-lico/Healthy Gut™ may provide relief for patients suffering from delayed GE.

## Discussion

Among the various factors and mechanisms involved in maintenance of gastric mucosal defense, there is the cyclooxygenase activity from COX-2, which synthesizes endogenous prostaglandin (PG). However, it is believed that an abundance of PG resulting from COX-2 activity due to inflammation can intensify inflammatory reactions in gastric mucosa and cause a deleterious effect (Kim et al. 2015).

Another vasoactive factor derived from vascular endothelium and/or produced by epithelial cells of the gastric mucosa is nitric oxide (NO), also involved in the mechanism of maintaining gastric integrity, protection and damage healing. NO in mucosa is formed by the activity of NO synthase (NOS) (Kwiecien et al. 2008) with the inducible NOS isoform (iNOS) known to synthesize NO in the mucosal epithelial cells of the stomach and the duodenum, and it has been associated with the inflammatory response initiated by infectious agents, toxic substances or stress (Barrachina et al. 2001). An excessive amount of NO released in inflammatory conditions leads to an increased interaction of NO with superoxide anion (O_2_^−^) resulting in the formation of a highly reactive peroxynitrite (ONOO^−^). These reactive species can cause cellular damage to phospholipids and DNA through oxidation (Pawlik et al. 2000; Ding et al. 2${\text{O}}_{2}^{-}$005).

In this study, AD-lico/Healthy Gut™ prevented the increased expression of COX-2 and iNOS in a dose-dependent manner in the epithelial cells following infection with *H. pylori*, and possibly explaining the mechanism of the reduced mucosal damage (Figures 1 and 2). The improvement in the *H. pylori*-induced damage in Figure 1 may be due to inhibition of pro-inflammatory reactions from the bacterial infection rather than preventing infection itself, although we did not demonstrate this. It is also assumed that AD-lico/Healthy Gut™ used a similar mechanism for reduction of pro-inflammatory mediators in the mucosal damage from water stress in the rat model (Figure 3).

Dietary supplements containing licorice as one of the key ingredients have also shown considerable efficacy in patients with FD. A meta-analysis of double-blind, randomized, clinical trials on a polyherbal combination containing licorice (Iberogast) demonstrated excellent overall therapeutic effect in treatment of FD. The findings showed a substantial improvement of symptoms with Iberogast but with varying superiority to placebo for the dyspepsia-specific gastrointestinal symptom scores. It should be noted that there is no single therapy available that is capable of providing relief to the majority of FD patients due to the pathophysiological heterogeneity of the disease, and there are several drugs approved or under development that include prokinetics and fundic relaxant drugs. Prokinetics include cisapride and mosapride, drugs that stimulate smooth muscle contraction to enhance GE and intestinal transit (Galligan and Vanner 2005) (Hiyama et al. 2007).

Impaired gastric accommodation in FD may contribute to symptom generation (Kindt and Tack 2006), and Matsueda et al. showed that acotiamide, a fundus-relaxing drug blocking the M1/M2 muscarinic receptor, significantly reduced the symptoms of FD (Matsueda et al. 2010). Our results with AD-lico/Healthy Gut™ in improving GE in rats are also indicative of the benefits of licorice components in improving stomach comfort. The AD-lico/Healthy Gut™ preparation of maximal levels of phenolic compounds, being low in component glycyrrhizin, may thus provide similar or superior benefits to other licorice-extracted drugs in terms of potency and side effects.
